# Epidemiology of orbital diseases in a tertiary ophthalmic outpatient clinic in Sao Paulo, Brazil

**DOI:** 10.5935/0004-2749.2024-0278

**Published:** 2025-02-13

**Authors:** Lissa Beltrão Fernandes, Marina Brandão Schmidt, Mário L. R. Monteiro, Allan C. Pieroni Gonçalves

**Affiliations:** 1 Division of Ophthalmology, Faculdade de Medicina, University de São Paulo, São Paulo, SP, Brazil.

**Keywords:** Orbital diseases, Orbital tumors, Neoplasms, Inflammation, Graves’ ophthalmopathy, Outpatients

## Abstract

**Purpose:**

This study aimed to evaluate the prevalence of orbital conditions in a tertiary ophthalmic outpatient hospital in Sao Paulo, Brazil, with a focus on the main diagnoses and their distribution.

**Methods:**

A retrospective chart review was conducted involving patients registered and admitted to the orbital disease unit at the Department of Ophthalmology, University of São Paulo Medical School, from January 2004 to March 2018. A total of 838 medical charts were analyzed, of which 37 were excluded due to incomplete data. The remaining charts were categorized into eight diagnostic groups: Graves’ orbitopathy , inflammatory disorders, tumors, vascular lesions, acquired structural abnormalities, congenital structural abnormalities, infectious diseases, and others.

**Results:**

Of the 837,300 ophthalmological appointments, 3,372 (0.4%) were related to orbital diseases. The study included 801 patients, of whom 63.45% were women. The patients’ mean age was 42.86 years. Graves’ orbitopathy was the most common (55%), followed by tumor (17%), inflammatory disorders (9%), vascular lesions (7%), acquired structural abnormalities (5%), congenital structural abnormalities (4%), others (2%), and infectious diseases (1%). The study found significant differences in the incidence and types of orbital diseases, indicating the specialized nature of tertiary care and referral biases.

**Conclusions:**

Published data on epidemiological orbital diseases is scarce. Therefore, this study focused on the diverse nature of orbital diseases and their low incidence among ophthalmology appointments. The major trends align with other epidemiological studies, demonstrating **a preponderance of Graves’ orbitopathy in middle-aged adults and a bimodal distribution of tumors. These findings are essential in shaping resident training programs and healthcare policies, particularly** in tertiary settings. Understanding the epidemiology of orbital diseases can improve diagnostic accuracy, treatment approaches, and patient outcomes as well as support future systemic prospective studies.

## INTRODUCTION

Orbital diseases encompass a diverse group of conditions that frequently pose diagnostic dilemma. The wide variety corresponds to the presence of multiple anatomical structures, originating from all three embryonic layers. Owing to their potential to cause severe cosmetic problems, visual loss, eye movement disorders, and eventually mortality, specialized and multidisciplinary attention is required. So far, the available epidemiological data in the literature regarding the frequency and distribution of these conditions is limited. Furthermore, the reported data of orbital diseases vary between studies, mainly depending on the source and geographical location of the examined material^(1^,^2)^. Such information is useful for shaping resident training programs and, on a larger scale, healthcare policies^(3)^.

This study aimed to evaluate the prevalence of orbital conditions in a tertiary ophthalmic outpatient hospital in Sao Paulo, Brazil, highlighting the main diagnoses and their demographic factors.

## METHODS

This study was approved by the Institutional Review Board. A retrospective chart review was conducted involving all registered and admitted patients in the orbital disease unit at the Department of Ophthalmology, Faculty of Medicine of the University of Sao Paulo, from January 2004 July to March 2018. A total of 838 medical charts were reviewed to obtain information on the age, gender, and diagnoses of the patients. The diagnoses were divided into eight categories: inflammatory disorders, Graves’ orbitopathy (GO), tumors, vascular lesions, acquired structural abnormalities, congenital structural abnormalities, infectious diseases, and others. Moreover, the total number of patients who attended (not necessarily admitted) the department of ophthalmology and the orbital disease unit over the same period was determined to calculate the overall frequency of orbital diseases.

## RESULTS

During the 14-year study period, 837,300 appointments were registered in the Department of Ophthalmology, with an average of 5,266 appointments per month. Orbital diseases accounted for 0.41% (n=3,472) of the total appointments.

Of the 838 patients admitted to the orbital disease unit, 37 were excluded due to incomplete data. Thus, only 801 patients were included in the final analysis.

**Figure 1:**
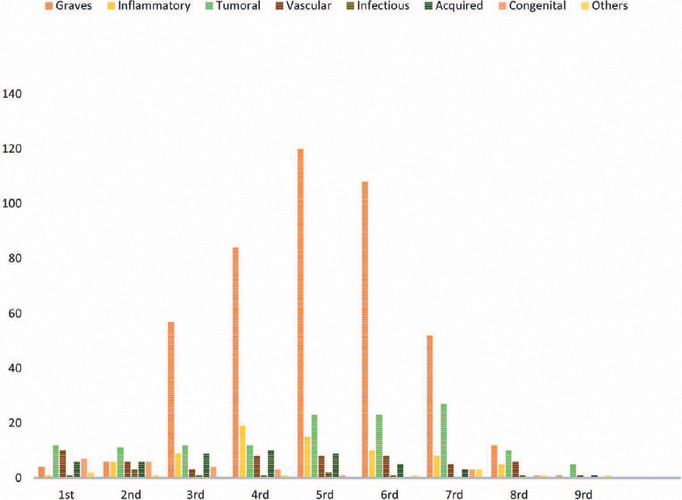
Histogram showing the distribution of orbital diseases per age by decade.

Among the patients, 63.45% (n=540) were women and 36.55% (n=311) were men. The orbital disease distribution per decade is illustrated in the histogram (Figure 1). The patients’ mean age was 42.86 (range, 0.2-91) years, with a median of 44 years.

**Figure 2:**
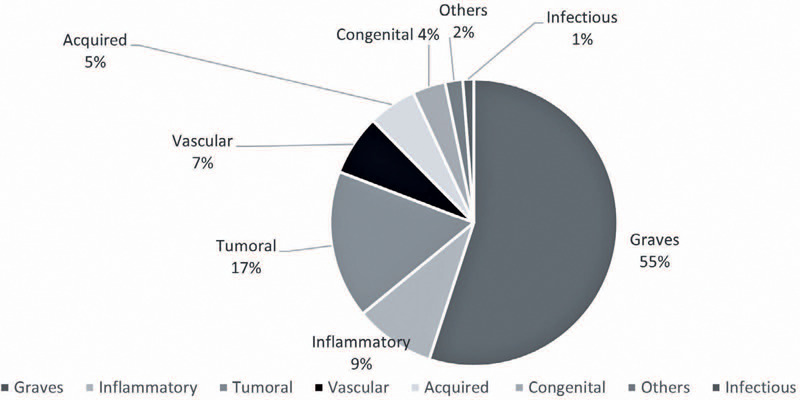
Pie chart showing the prevalence of each diagnostic category of the orbital diseases unit.

The pie chart (Figure 2) shows the overall prevalence of the eight diagnostic categories. GO was the most common (55%), followed by tumors (17%), inflammatory disorders (9%), vascular lesions (7%), acquired structural abnormalities (6%), congenital structural abnormalities (4%), others (2%), and infectious diseases (1%). Table 1 presents the most prevalent diseases in each category and their incidence rate. Table 2 outlines the most prevalent lesions in the tumor category in childhood (<20 years) and adulthood (>20 years).

**Table 1:** Breakdown of orbital affections prevalence in each category, excluding Graves’ orbitopathy

Orbital diseases			
Category	Most prevalent affections		
Inflammatory disorders	Idiopathic 76.7%	lgG4 disease 5.5%	Others 15%
Tumors	Lymphoma 17%	Meningioma 8.9%	Metastasis 8.1%
Vascular lesions	Lymphangioma 36%	Cavernous hemangioma 34.5%	Carotid-cavernous fistula 20%
Infectious diseases	Orbital abscess 60%	Fungal infections 30%	Osteomyelitis 10%
Acquired structural abnormalities	Fractures 81.8%	Mucocele 13.6%	Fat prolapses 2.3%
Others	Pseudoproptosis 40%	Progressive ophthalmoplegia 10%	Miscellaneous 50%
Congenital structural abnormalities	Dermoid cyst 26.7%	Epidermoid cyst 26.7%	Dermolipoma 13.3%

**Table 2:** Tumoral category divided by childhood and adulthood

Childhood (<20 years)		Adulthood (>20 years)	
Capillary hemangioma	21.7%	Lymphoma	20.5%
Glioma	13%	Lacrimal gland tumor - Pleomorphic adenoma 47.05% - Pleomorphic adenocarcinoma 29.41% - Adenoid cystic carcinoma 5.88%	15.2%
Rhabdomyosarcoma	13%	Meningioma	10.7%
Fibrous dysplasia	8.7%	Metastasis	8.9%

## DISCUSSION

Our study was conducted in a single tertiary referral hospital located in the largest metropolitan area of South America and known for its diverse, multiracial population. The overall frequency of orbital diseases observed in our study, at 0.4%, closely resembled^(2^,^5^,^6)^.

So far, epidemiological studies are scarce, and existing ones demonstrate significant differences in frequencies and age distributions between the different types of diseases^(1^,^3^,^7)^. This variation is thought to be caused by several factors, including regional population differences, referral biases, focus on neoplastic or non-neoplastic conditions, and the authors’ chosen nosology and classification systems. In addition, the involvement of various specialties in orbital disease treatment may influence the data, depending on the practices of the hospital.

### Graves’ orbitopathy (GO)

In the fourth to sixth decades of life, GO accounted for 70.2% of our cases (Figure 1), consistent with that in other studies^(2^,^8^,^9^,^10)^. However, some studies reported lower rates of GO prevalence in middle age^(6)^.

Among the young patients (≤20 years) in this study, 11% had GO. Other studies reported GO as an uncommon event in patients aged below 20 years, with lower frequency rates of approximately 4% of the orbital diseases^(6^,^11)^.

Most of the GO patients were women, with the female-to-male ratio being 3.2:1.0. Other studies involving distinct populations reported higher ratios, i.e., 5.1:1.0^(8^,^12)^ and 5.2:1.0^(13)^. Conversely, a lower ratio of 2.1:1.0 was reported in a study involving a higher number of mild cases^(14)^. In our study, the higher percentage of men was likely due to referral bias as they tend to require complex GO management through multiple-stage procedures.

### Inflammatory disorders

Consistent with other reports, orbital inflammatory diseases accounted for up to 9% of our cases, equally affecting all ages and both genders^(6^,^7^,^15)^.

Within this diagnostic category, idiopathic inflammatory orbital (IIO) disease was the most common condition, accounting for 69.73% of the cases. Similar results have been reported in the literature, with one study reporting a prevalence of 74%, although infectious diseases were included in that group^(7)^. As IIO is a diagnosis of exclusion, other pathological conditions affecting the orbit must be ruled out. With the increase in the understanding of the pathology, lgG4-related disease accounts for a considerable portion of cases previously diagnosed as idiopathic inflammation or reactive lymphoid hyperplasia^(16)^.

The requirement for orbital biopsy is carefully considered. Some practitioners prefer to initially perform biopsy, whereas others reserve it for refractory cases. Depending on the standard diagnostic procedures of each service, this can interfere with the percentage of nonspecific inflammatory diseases and others, such as lgG4-related disease. Furthermore, previous use of corticosteroids may lead to false-negative biopsy results.

Serological studies have limited value in the diagnosis of nonthyroid orbital inflammation as they typically require a considerable degree of disease progression to yield a positive result. In addition, the findings from these tests are often nonspecific, making it difficult to definitively diagnose the condition based on serology alone^(17)^.

### Tumors

Orbital tumors encompass a group of diverse lesions, each demonstrating distinct characteristics but collectively showing a low incidence rate in the population.

In our study, a high number of tumoral lesions (30 cases, accounting for 19% of our cases) had undetermined etiology. This was possibly caused by several factors, such as socioeconomic adversity, referral to systemic oncologic care, and deterioration of patients’ general health in cases of systemic neoplasia. These issues often resulted in inadequate follow-up, leading to incomplete or unavailable medical records.

In younger age groups, the most common malignant tumor was rhabdomyosarcoma, consistent with the findings in other studies^(2^,^6^,^18)^. In older patients, lymphoma predominated, as reported in several studies^(2^,^5^,^19)^. The increasing incidence of lymphoma could be partially attributed to the use of new diagnostic methods that more accurately distinguish low-grade lymphomas, which were previously diagnosed as pseudolymphoma or IIO disease.

Meningioma is reportedly the most common benign tumor in adulthood, as in our study^(1^,^7^,^19)^ (Table 2).

Lacrimal gland lesions are projected to have a low incidence^(1)^, which could be due to the exclusion of lymphomas from lacrimal gland tumors. Nevertheless, benign epithelial tumors remain prevalent, consistent with the finding in most studies, comprising half of all epithelial tumors.

Dacryops, which typically represents the highest in-cidence^(7)^, due to its simple management, had no cases admitted to our tertiary care unit. Other studies reported the incidence of the following epithelial lacrimal gland tumors: pleomorphic adenoma, adenoid cystic carcinoma, pleomorphic adenocarcinoma, and adenocarcinoma. However, our study yielded different results, which indicated a higher rate of pleomorphic adenocarcinoma (Table 2)^(1^,^7^,^20^,^21)^. Pleomorphic adenocarcinoma may arise *de novo* or more commonly as a recurrence of a previous inadequately resected adenoma, which could reflect our limited experience as a developing country.

We found that metastatic and secondary invading tumors accounted for 8.1% of all tumors. Most longdistance metastatic tumors originated from the breast and lymphoma.

The discussed differences indicate geographical variation in the frequency of various tumors. Such a variation may be due to the overrepresentation of certain tumors related to specific age groups in our tertiary care hospital. Moreover, orbital tumors constitute a group of diverse lesions with a low incidence in the population, suggesting that a single case can substantially impact percentage variation.

Previous authors have reported that these factors contribute to the observed discrepancies in tumor frequencies^(22)^.

Capillary infantile hemangioma (i.e., benign hemangioendothelioma) was classified as a tumor lesion and was the most prevalent orbital vascular tumor in childhood, accounting for 5.6% of our cases.

### Vascular lesions

Vascular lesions accounted for approximately 7% of all orbital pathologies in our study. As the exact nature of cavernous malformations remains unclear, we categorized them as vascular lesions according to the consensus of the Orbital Society^(23)^. However, some authors asserted that these lesions are of venous origin, whereas others have proposed that they represent low-flow arteriovenous malformations.

Lymphangioma, accounting for 36% of the cases, and cavernous hemangioma, accounting for 34%, had the highest prevalence, followed by carotid-cavernous fistula at 20%. Consistent with the literature findings, lymphangioma is commonly diagnosed in the first two decades of life, whereas cavernous hemangioma predominates in adulthood^(1^,^7)^.

### Acquired structural abnormalities

Traumatic orbital fracture was the most common in this category, accounting for only 6% of the orbital conditions in our study, contrary to other studies in which it accounted for 27.5% of the oculoplastic conditions^(3)^. These discrepancies could be attributed to referral bias. Cases of facial and orbital traumas are typically admitted to the emergency department, where oral maxillofacial and craniomaxillofacial teams are more readily available than ophthalmic orbital specialists. Furthermore, another relevant acquired structural abnormality, mucocele, is referred to the department of otolaryngology in our hospital.

### Congenital structural abnormalities

The most common congenital structural abnormalities in our study were cystic lesions, including dermoid and epidermoid cysts, which accounted for 53.33% of the cases and represented 2% of general orbital diseases. Despite expectations of a higher prevalence, as observed in other studies (8.3-12.1%)^(2^,^24)^, our tertiary position reduces the number of noncomplex diagnoses and treatments.

Following cystic lesions, fibrous dysplasia accounted for 16.6% of the congenital structural abnormalities. The literature varies in terms of the frequency of fibrous dysplasia, ranging from “rare” (5 out of 764 cases of orbital tumors at the Mayo Clinic over 26 years) to “not uncommon” (144 cases of fibrous dysplasia of the skull).

Large-scale studies reported a lower incidence of microphthalmia with orbital cyst and lacrimal gland cyst, consistent with the present study^(1^,^4)^.

### Infectious diseases

In our study, abscesses were the most frequent clinical presentation in the group of infectious disease, accounting for 40% of the cases. Periorbital cellulitis had a higher incidence (83%) than orbital cellulitis^(4)^.

Cellulitis is typically diagnosed and treated in emergency units or primary or secondary care settings. Cases referred to an orbital service are usually more complex, having worse evolution or requiring specialized diagnoses or treatment. Notably, fungal etiology was present in almost one-third of the cases, which is an atypical and rare finding in nontertiary practices.

This study highlights the diverse nature of orbital diseases and the substantial geographical and institutional variations in terms of incidence and types. Although some variations exist, the major trends are consistent between our study and other epidemiological studies: a preponderance of GO in middle-aged adults, a bimodal distribution of tumors, a gradual decrease in structural abnormalities with age, and a relatively uniform occurrence of inflammatory and vascular diseases. Conversely, other findings, such as the higher incidence of pleomorphic adenocarcinoma and the unique distribution of infectious diseases, reflect the specialized nature of tertiary care and referral biases. The frequencies of various orbital malignancies demonstrate geographical variation in both pediatric and adult populations.

The low overall incidence of orbital diseases (0.4%) among the ophthalmology appointments indicates the rarity and complexity of these conditions, which require specialized multidisciplinary approaches for an effective management.

These findings are crucial for shaping resident training programs and healthcare policies, particularly in tertiary settings, and for building a framework to analyze orbital diseases. Understanding the epidemiology of orbital diseases can contribute to the improvement of diagnostic accuracy, treatment approaches, and patient outcomes, resulting in better healthcare delivery for these challenging conditions. Moreover, our findings can be correlated with future systemic prospective studies.

## References

[1] Johansen S, Heegaard S, Bøgeskov L, Prause JU. (2000). Orbital space-occupying lesions in Denmark
1974-1997. Acta Ophthalmol Scand.

[2] Rootman J. (2003). Diseases of the orbit : a multidisciplinary approach.

[3] Tan MC, Young S, Amrith S, Sundar G. (2012). Epidemiology of oculoplastic conditions: the Singapore
experience. Orbit.

[4] Assavedo CR, Monteiro S, Kinkpe E, Sounouvou I, Tchab Hounnou S, Doutetien Gbaguidi C. (2022). Epidemiological, clinical and therapeutic aspects of orbital
diseases in Ophthalmologic Hospital of Saint André de Tinré
(OHSAT), in Benin Republic: a retrospective study. Issues and Developments in Medicine and Medical Research B P
International.

[5] De Concilis C., Bosniak S (1996). Principles and practice of ophthalmic plastic and reconstructive
surgery.

[6] Montoya FJ, Perez-Moreiras JV., Perez-Moreiras JV, Prada-Sánches MC (2004). Orbit.

[7] Shields JA, Shields CL, Scartozzi R. (2004). Survey of 1264 patients with orbital tumors and simulating
lesions: The 2002 Montgomery Lecture, part 1. Ophthalmology.

[8] Ackuaku-Dogbe EM, Akpalu J, Abaidoo B. (2017). Epidemiology and Clinical Features of Thyroid-associated
Orbitopathy in Accra. Middle East Afr J Ophthalmol.

[9] Bodh SA, Goel R, Kumar S, Bansal S, Singh M. (2012). Thyroid associated ophthalmopathy. Delhi J Ophthalmol.

[10] Yeatts RP. (2005). Quality of life in patients with Graves
ophthalmopathy. Trans Am Ophthalmol Soc.

[11] Krassas GE, Gogakos A. (2006). Thyroid-associated ophthalmopathy in juvenile Graves’
disease-clinical, endocrine and therapeutic aspects. J Pediatr Endocrinol Metab.

[12] Sarfo-Kantanka O, Sarfo FS, Ansah EO, Kyei I. (2018). Graves Disease in central ghana: clinical characteristics and
associated factors. Clin Med Insights Endocrinol Diabetes.

[13] Bartley GB, Fatourechi V, Kadrmas EF, Jacobsen SJ, Ilstrup DM, Garrity JA (1995). The incidence of Graves’ ophthalmopathy in Olmsted County,
Minnesota. Am J Ophthalmol.

[14] Reddy SV, Jain A, Yadav SB, Sharma K, Bhatia E. (2014). Prevalence of Graves’ ophthalmopathy in patients with Graves’
disease presenting to a referral centre in north India. Indian J Med Res.

[15] Gordon LK. (2006). Orbital inflammatory disease: a diagnostic and therapeutic
challenge. Eye (Lond).

[16] Yu WK, Tsai CC, Kao SC, Liu CJ. (2018). Immunoglobulin G4-related ophthalmic disease. Taiwan J Ophthalmol.

[17] Mombaerts I, Rose GE, Garrity JA. (2016). Orbital inflammation: biopsy first. Surv Ophthalmol.

[18] Hassan WM, Bakry MS, Hassan HM, Alfaar AS. (2016). Incidence of orbital, conjunctival and lacrimal gland malignant
tumors in USA from Surveillance, Epidemiology and End Results,
1973-2009. Int J Ophthalmol.

[19] Ting DS, Perez-Lopez M, Chew NJ, Clarke L, Dickinson AJ, Neoh C. (2015). A 10-year review of orbital biopsy: the Newcastle Eye Centre
Study. Eye (Lond).

[20] Seregard S, Sahlin S. (1999). Panorama of orbital space-occupying lesions. The 24-year
experience of a referral centre. Acta Ophthalmol Scand.

[21] Günalp IG. (2009). Biopsy-proven orbital lesions in Turkey: A survey of 1092 cases
over 30 years. Int J Orbital Dis. Oculoplast Lacr Surg.

[22] Smoker WR, Gentry LR, Yee NK, Reede DL, Nerad JA. (2008). Vascular lesions of the orbit: more than meets the
eye. Radiographics.

[23] Harris GJ, Orbital Society (1999). Orbital vascular malformations: a consensus statement on
terminology and its clinical implications. Am J Ophthalmol.

[24] Henderson JW, Farrow GM, Garrity JA. (1994). Orbital Tumors.

